# Supplementary material for: Presenting Symptoms in Newly Diagnosed Myeloma, Relation to Organ Damage, and Implications for Symptom-Directed Screening: A Secondary Analysis from the Tackling Early Morbidity and Mortality in Myeloma (TEAMM) Trial
Source: Cancers (Basel). 2023 Jun 25;15(13):3337. doi: 10.3390/cancers15133337 (PMC10341254; doi:10.3390/cancers15133337)
Supplement: Supplementary file 1 [file cancers-15-03337-s001.zip › cancers-2283587-supplementary.docx]

**Supplementary data**

**Presenting symptoms in newly diagnosed myeloma, relation to organ damage and implications for symptom-directed screening: A secondary analysis from the Tackling EArly Morbidity and mortality in Myeloma (TEAMM) trial**

**Table S1 Eligibility criteria**

| **Inclusion criteria** |
| --- |
| • Age ≥ 21 years and able to give informed consent  • Patient with newly diagnosed symptomatic myeloma based on internationally agreed criteria  • There is an intention to treat the patient’s myeloma actively  • Patient that is no more than 14 days prior to or no more than 14 days into starting a programme of antimyeloma therapy  • Provision of written informed consent |
| **Exclusion criteria** |
| Patients with contraindication to Levofloxacin:-  ♣ known to have sensitivity / allergy to Levofloxacin or other quinolones  ♣ Patients with a history of tendon disorders related to fluoroquinolone administration  ♣ Patients receiving other antibacterial prophylaxis (excluding pneumocystis prophylaxis if regarded as essential)  ♣ Patients receiving amiodarone or arsenic trioxide  ♣ Patients on active antiepileptic treatment  • Women of childbearing age who are not willing to use appropriate methods of contraception to prevent pregnancy or women that are breastfeeding  • Patient thought to have mandatory requirement for antibacterial prophylaxis  • Previous treatment for myeloma, except for the following:  ♣ Local radiotherapy to relieve bone pain or spinal cord compression  ♣ Prior bisphosphonate treatment  • Previous (<5 years since diagnosis) or concurrent active malignancies except surgically removed basal or squamous cell carcinoma of the skin, treated carcinoma in situ of the breast or cervix, or incidental histologic finding of prostate cancer (TNM stage of T1a or 1b). Previous (>5 years) of other cured malignancies may be entered. |

**Table S2. Symptom questions asked of patients at trial entry**

| With hindsight, what were the first symptoms that we can now attribute to myeloma and when did they occur?  After the first 197 participants this was amended to:  When did the patient first notice bodily changes and/or symptoms that they attribute to the myeloma?’ to ensure agreement with the newly published Aarhus statement.^18^  The question included four lines to record the symptoms, plus date of onset for each line. The other questions asked were as follows:  1. When did the patient first visit a doctor or nurse at their local general practice about any of these symptoms or bodily changes?  2. How many times did the patient consult a doctor or nurse at their local general practice about any of these symptoms or bodily changes before they were referred (or diagnosed, if no general practitioner/primary care physician referral)?  3. When did the patient first visit the hospital (includes accident and emergency department)?  4. Which hospital department was the patient first seen in (includes accident and emergency)?  5. When did the patient first see a haematologist about any of these symptoms? |
| --- |

**Table S3.** Total symptoms/reasons for referral reported by the time of active MM diagnosis**.** The broad categories are in bold, with the numbers of patients reporting individual symptoms within each category below the broad category heading. Patients could report more than one symptom. **AKI Acute kidney injury.** CKD Chronic kidney disease. *symptoms of spinal cord compression is a composite of: stated spinal cord compression (2), leg weakness (8), leg numbness/altered sensation (6), incontinence (5), arm numbness/altered sensation (3), arm weakness (1). DVT Deep vein thrombosis, PE Pulmonary embolus.

| **Back pain**  **Including neck pain** | **364** | **Self-reported anaemia** | **113** | **Abnormal blood results – myeloma related**  **Hypercalcaemia**  **Raised total protein**  **Raised ESR** | **51**  **34**  **10**  **7** | **Bleeding or thrombosis**  **Epistaxis**  **Haematuria**  **Bruising**  **Haemoptysis**  **PE or DVT**  **Gum bleeding** | **25**  **12**  **5**  **3**  **2**  **2**  **1** |
| --- | --- | --- | --- | --- | --- | --- | --- |
| **Systemic symptoms**  Fatigue  Weight loss  Generally unwell  Anorexia  Sweats (including night sweats)  Collapse/dizziness  Reduced exercise tolerance  Falls  Low mood  Weight gain | **335**  155  89  23  21  16  12  11  6  1  1 | **Gastrointestinal (GI) symptoms**  Abdominal pain  Vomiting  Nausea  Constipation  Diarrhoea  Altered bowel habit  Dyspepsia  GI bleeding  Dysphagia  Jaundice  Unspecified ‘GI symptoms’ | **86**  35  15  12  7  5  3  3  3  1  1  1 |  |  |  |  |
|  |  |  |  | **Renal**  Unspecified  AKI  CKD  AKI on CKD  Proteinuria  Post-operative renal failure  Renal failure  Worsening function | **46**  26  12  3  1  1  1  1  1 | **Other symptoms**  Limb swelling  Rash/skin problems  Mouth ulcers/mucositis  Atrial fibrillation  Hypertension  Itching  Pallor  Palpitations  Restless legs | **23**  11  3  2  1  1  2  1  1  1 |
|  |  |  |  | **Bone disease**  Fracture  Lytic lesion(s)  Unspecified  Osteoporosis | **45**  34  7  3  1 |  |  |
|  |  | **Respiratory symptoms**  Shortness of breath  Cough  Hoarse voice  Abnormal chest x-ray  Non-specific respiratory symptom | **78**  62  12  2  1  1 | **Abnormal blood results - non-specific**  Unspecified abnormal blood result  Neutropenia  Pancytopenia  Thrombocytopenia  B12 deficiency  High PSA  Hyperkalemia  Hypernatremia  Hyperparathyroidism  Hypogammaglobulinemia  Leucopenia  Low albumin  Low total protein  Raised ferritin  Raised inflammatory markers  Thrombocytosis | **32**  10  5  2  2  1  1  1  1  1  2  1  1  1  1  1  1 | **Abnormal lumps**  Chest swelling  Lymphadenopathy  Neck lump  Clavicle lump  Head lump  Lower back lump  Scapula  Unspecified lump | **12**  3  2  2  1  1  1  1  1 |
| **Other pain symptoms**  Chest pain (including rib pain)  General pain  Limb pain (hip)  Limb pain (shoulder)  Limb pain (leg)  Limb pain (arm)  Joint pain  Joint pain (knee)  Limb pain (groin)  Face pain  Foot pain  Hand pain  Limb pain  Radicular pain | **325**  97  73  53  39  21  14  9  7  5  2  2  1  1  1 | **Infection**  Lower respiratory infection  UTI  Fever  ‘Recurrent’ (site not specified)  Upper respiratory infection  Cellulitis  ‘Viral’  Tooth | **59**  30  7  7  6  2  1  1  1 |  |  |  |  |
|  |  | **Neurological symptoms**  Symptoms of spinal cord compression*  Headache  Confusion  Visual disturbance  Carpal tunnel | **54**  25  14  8  5  2 |  |  | **Urological symptoms**  Urinary frequency  Bladder outflow problems  Nocturia  Reduced urine output  Urgency | **8**  3  2  1  1  1 |

**Table S4. Fractures reported on case report forms at trial entry taken from imaging reports.** Patients could have more than one fracture.

| Site of fracture | Number of patients |
| --- | --- |
| Vertebral | 270 |
| Rib | 56 |
| Arm | 15 |
| Hip/NOF | 11 |
| Clavicle | 7 |
| Pubic rami/pelvis | 5 |
| Acromion | 1 |
| Mandible | 1 |
| Sternum | 1 |
| Thumb | 1 |

| Baseline parameters | | | Back pain | | No back pain | | P* | | Other pain | | No other  pain | | P* | | Systemic symptoms | | No systemic  symptoms | | P* | |
| --- | --- | --- | --- | --- | --- | --- | --- | --- | --- | --- | --- | --- | --- | --- | --- | --- | --- | --- | --- | --- |
|  | | | N (%) | | N (%) | |  | | N (%) | | N (%) | |  | | N (%) | | N (%) | |  | |
| Number of patients (%) | | | 364 (38) | | 598 (62) | |  | | 295 (31) | | 667 (69) | |  | | 265 (28) | | 697 (73) | |  | |
| Age (years) | Median  IQR | 66  60-73 | | 68  60-76 | | >0.99 | | 66  58-73 | | 68  60-75 | | 0.84 | | 67  59-75 | | 67  60-75 | | >0.99 | |  |
| Ethnicity | White | 326 (90) | | 550 (92) | | >0.99 | | 267 (91) | | 609 (91) | | >0.99 | | 237 (89) | | 639 (92) | | >0.99 | |  |
| Gender | Male | 228 (63) | | 373 (62) | | >0.99 | | 183 (62) | | 418 (63) | | >0.99 | | 160 (60) | | 441 (63) | | >0.99 | |  |
| Planned high intensity treatment | Yes | 210 (58) | | 313 (52) | | >0.99 | | 181 (61) | | 342 (51) | | 0.21 | | 145 (55) | | 378 (54) | | >0.99 | |  |
| PS 6 m before | 0-1  2-4  Unavailable | 334 (92)  23 (6)  7 (2) | | 544 (90)  27 (5)  27 (5) | | >0.99 | | 268 (91)  15 (5)  12 (4) | | 610 (91)  35 (5)  22 (3) | | >0.99 | | 250 (94)  9 (3)  6 (2) | | 628 (90)  41 (6)  28 (4) | | >0.99 | |  |
| PS at entry | 0-1  2-4  Unavailable | 245 (67)  116 (32)  3 (1) | | 479 (80)  104 (17)  15 (3) | | 0.004 | | 216 (73)  72 (24)  7 (2) | | 508 (76)  148 (22)  11 (2) | | >0.99 | | 203 (77)  59 (22)  3 (1) | | 521 (75)  161 (23)  15 (2) | | >0.99 | |  |
| Fall in PS | No change  Deteriorated  Missing | 176 (48)  181 (50)  7 (2) | | 360 (60)  210 (35)  28 (5) | | 0.004 | | 152 (52)  130 (44)  13 (4) | | 384 (58)  261 (39)  22 (3) | | >0.99 | | 137 (52)  122 (46)  6 (2) | | 399 (57)  269 (39)  29 (4) | | >0.99 | |  |
| ISS | 1  2  3  Unavailable | 86 (24)  133 (37)  87 (24)  58 (16) | | 127 (21)  217 (36)  160 (27)  94 (16) | | >0.99 | | 70 (24)  110 (37)  68 (23)  47 (16) | | 143 (21)  240 (36)  179 (27)  105 (16) | | >0.99 | | 45 (17)  91 (34)  88 (33)  41 (15) | | 168 (24)  259 (37)  159 (23)  111 (16) | | 0.21 | |  |
| Hypercalcaemia | Yes  No  Unavailable | 19 (5)  265 (73)  80 (22) | | 26 (4)  443 (74)  129 (22) | | >0.99 | | 16 (5)  211 (72)  68 (23) | | 29 (4)  497 (75)  141 (21) | | >0.99 | | 15 (6)  181 (68)  69 (26) | | 30 (4)  527 (76)  140 (20) | | >0.99 | |  |
| Renal impairment | Yes  No  Unavailable | 36 (10)  325 (89)  3 (1) | | 112 (19)  475 (79)  11 (2) | | 0.17 | | 32 (11)  257 (87)  6 (2) | | 116 (17)  543 (81)  8 (1) | | 0.84 | | 55 (21)  207 (78)  3 (1) | | 93 (13)  593 (85)  11 (2) | | 0.84 | |  |
| Anaemia | Yes  No  Unavailable | 149 (41)  143 (39)  72 (20) | | 250 (42)  236 (39)  112 (19) | | >0.99 | | 110 (37)  127 (43)  58 (20) | | 289 (43)  252 (38)  126 (19) | | >0.99 | | 135 (51)  70 (26)  60 (23) | | 264 (38)  309 (44)  124 (18) | | 0.004 | |  |
| Bone disease | Yes  No  Unavailable | 313 (86)  48 (13)  3 (1) | | 367 (61)  227 (38)  4 (1) | | 0.004 | | 239 (81)  51 (17)  5 (2) | | 441 (66)  224 (34)  2 (<1) | | 0.004 | | 172 (65)  93 (35)  0 (0) | | 508 (73)  182 (26)  7 (1) | | 0.29 | |  |
| Vertebral fracture | Yes | 179 (49) | | 91 (15) | | 0.004 | | 71 (24) | | 199 (30) | | 0.004 | | 56 (21) | | 214 (31) | | 0.21 | |  |
| Number of CRAB features | 0  1  2  3  Unavailable | 15 (4)  131 (36)  106 (29)  29 (8)  83 (23) | | 54 (9)  224 (37)  136 (23)  44 (7)  140 (23) | | 0.84 | | 13 (4)  121 (41)  66 (22)  22 (7)  73 (25) | | 56 (8)  234 (35)  176 (26)  51 (8)  150 (22) | | >0.99 | | 15 (6)  80 (30)  74 (28)  26 (10)  70 (26) | | 54 (8)  275 (39)  168 (24)  47 (7)  153 (22) | | >0.99 | |  |

**Table S5: Major symptom groups in relation to prognostic factors and CRAB features at trial entry** (*p-values with Bonferroni correction for multiple testing. **42**

**Table S6: Broad symptom profile in relation to potentially irreversible organ damage (renal failure and imaging reported vertebral fractures)**

|  | **Participants reporting one or more symptoms in each category** | | | | | | | | | | | |
| --- | --- | --- | --- | --- | --- | --- | --- | --- | --- | --- | --- | --- |
| **Symptom categories** | **Renal failure**  **eGFR<40**  **(n=148)** | | **No renal failure**  **eGFR ≥40**  **(n=800)** | | | |  | **Vertebral**  **Fracture**  **(n=270)** | | **No vertebral**  **fracture**  **(n=692)** | |  |
|  | **N** | **%** | | **N** | **%** | **P^+^** | | **N** | **%** | **N** | **%** | **P^+^** |
| Back pain | 36 | 24 | | 325 | 41 | 0.007 | | 179 | 66 | 185 | 27 | 0.002 |
| Other pain *(excluding back pain)* | 32 | 22 | | 257 | 32 | 0.22 | | 71 | 26 | 224 | 32 | >0.99 |
| Systemic symptoms | 55 | 37 | | 207 | 26 | 0.15 | | 56 | 21 | 209 | 30 | 0.09 |
| Self-reported anaemia | 17 | 11 | | 94 | 12 | >0.99 | | 18 | 7 | 95 | 14 | 0.04 |
| Respiratory symptoms | 19 | 13 | | 53 | 7 | 0.22 | | 10 | 4 | 64 | 9 | 0.13 |
| Gastrointestinal symptoms | 22 | 15 | | 50 | 6 | 0.01 | | 16 | 6 | 57 | 8 | >0.99 |
| Infection | 5 | 3 | | 49 | 6 | >0.99 | | 8 | 3 | 46 | 7 | 0.88 |
| Neurological symptoms | 7 | 5 | | 45 | 6 | >0.99 | | 13 | 5 | 40 | 6 | >0.99 |
| Abnormal blood results (suggestive of myeloma) | 9 | 6 | | 42 | 5 | >0.99 | | 6 | 2 | 45 | 7 | 0.22 |
| Self-reported bone disease & fracture* | 1 | 1 | | 40 | 5 | 0.22 | | 19 | 7 | 23 | 3 | 0.44 |
| Self-reported renal problems | 28 | 19 | | 17 | 2 | 0.002 | | 8 | 3 | 38 | 5 | >0.99 |

*Note: Fracture patients were those where fracture (including vertebral fracture) were reported on baseline case report form. *denotes patients where they self-reported a fracture, lytic lesion or osteoporosis as reason for referral to secondary care.*  ^+^p-values with Bonferroni correction for multiple testing. **22**

977 participants

No data available for 15 participants

962 participants with data available

67 participants who did not describe any bodily symptoms but recorded laboratory results as a reason for referral or MGUS describe any bodily symptoms but recorded laboratory results as a reason for referral or MGUS

895 participants who recorded data describing symptoms

767 participants who recorded symptoms prior to first healthcare consultation

**Figure S1: Consort diagram showing participant responses to questions regarding symptoms related to multiple myeloma.**
